# Design and Optimization of Omeprazole-Curcumin-Loaded Hydrogel Beads Coated with Chitosan for Treating Peptic Ulcers

**DOI:** 10.3390/ph16060795

**Published:** 2023-05-27

**Authors:** Eman J. Heikal, Rashad M. Kaoud, Shadeed Gad, Hatem I. Mokhtar, Afaf A. Aldahish, Wafa Ali Alzlaiq, Sawsan A. Zaitone, Yasser M. Moustafa, Taha M. Hammady

**Affiliations:** 1 Department of Pharmaceutics and Industrial Pharmacy, Faculty of Pharmacy, Suez Canal University, Ismailia 41522, Egypt; 2 Faculty of Pharmacy, The University of Mashreq, Baghdad 10001, Iraq; 3 Pharmacy Department, Ashur University College, Baghdad 10047, Iraq; 4 Department of Pharmaceutical Chemistry, Faculty of Pharmacy, Sinai University-Kantara Branch, Ismailia 41636, Egypt; 5 Department of Pharmacology and Toxicology, College of Pharmacy, King Khalid University, Abha 62529, Saudi Arabia; 6 Department of Clinical Pharmacy, College of Pharmacy, Imam Abdulrahman Bin Faisal University, Dammam 31441, Saudi Arabia; 7 Department of Pharmacology and Toxicology, Faculty of Pharmacy, University of Tabuk, Tabuk 71491, Saudi Arabia; 8 Department of Pharmacology and Toxicology, Faculty of Pharmacy, Suez Canal University, Ismailia 41522, Egypt; 9 Department of Pharmacology and Toxicology, Faculty of Pharmacy, Badr University in Cairo, Cairo 11829, Egypt

**Keywords:** omeprazole, curcumin, hydroxypropyl-β-cyclodextrin, chitosan-alginate hydrogel, ulcer

## Abstract

This study aimed to formulate a pharmaceutical dosage form containing omeprazole (OMP) and curcumin (CURC) to treat experimental peptic ulcers. OMP and CURC were preliminarily complexed with hydroxypropyl-β-cyclodextrin for enhancing their solubilization. After that, the combined complex (CURC/OMP) was loaded to alginate beads to sustain their release and then coated with chitosan. Finally, we tested the anti-ulcerogenic impact of the best formula versus free OMP or OMP-only-loaded beads. The formulated spherical beads’ diameter ranged from a minimum value of 1.5 ± 0.08 mm to 2.6 ± 0.24 mm; the swelling results ranged from 400.00 ± 8.5% to 800.00 ± 6.2%. The entrapment efficiency was in a range from 60.85 ± 1.01% to 87.44 ± 1.88%. The optimized formula (F8) showed a maximum EE% (87.44 ± 1.88%), swelling (800.00 ± 6.2%), and diameter in the range of 2.60 ± 0.24, with a desirability of 0.941. In the first hour following the administration of the free drug complex, 95% of OMP and 98% of CURC were released. This is unacceptable for medications that require a delayed release in the stomach. The initial drug release from hydrogel beads was 23.19% for CURC and 17.19% for OMP after 2 h and 73.09% for CURC and 58.26% for OMP after 12 h; however, after 24 h, 87.81% of CURC and 81.67% of OMP had been released. The OMP/CURC beads showed a more stable particle size (0.52 ± 0.01 mm) after 6 weeks. In conclusion, the OMP/CURC hydrogel beads give stronger anti-ulcer effectiveness compared to free OMP, CURC-only beads, and OMP-only-loaded beads, indicating a prospective application for managing peptic ulcers.

## 1. Introduction

Peptic ulcer disease (PUD) and its sequelae have been recognized as the cause of many deaths per year. In high-income countries, mortality and morbidity from PUD have declined in recent years due to the use of medications targeting H. pylori. In fact, PUD continues to be a challenge in developing countries [1].

Defects in the gastric mucosa are arbitrated by the imbalance between the mucosal protective and damaging machineries, resulting in the generation of ulcers. Although H. pylori infection has been generally recognized as an essential factor in the pathogens of PUD, the interaction between etiological factors in the PUD pathology must be considered. This may include NSAID consumption, psychological stress, genetic factors, changed HCl secretion, defective mucosal defense mechanisms, and smoking. PUD consists of two types of ulcers, gastric and duodenal ulcers, but can also be found in the lower esophagus [2]. Hence, the effective control of gastric acid secretion is an essential fighter against PUD pathogenesis [3].

Omeprazole (OMP) is a potent inhibitor for gastric HCl secretion, binding irreversibly to the parietal cell proton pump (HK-ATPase), which is the direct gate of acid secretion, thus producing prolonged-lasting acid inhibition. Curcumin (CURC) is a polyphenol extracted from the rhizomes of turmeric (Curcuma longa Linn) with a documented propitious protective effect against peptic ulcer development [4]. CURC is known to attenuate gastric mucosal damage, not only through its antioxidant and anti-inflammatory properties but also by promoting wound healing and preventing apoptosis [5]. CURC is safe for oral administration [6]. Nevertheless, the poor aqueous solubility and short gastric residence time for CURC are considerable limitations for its use in PUD [7]. Further, OMP is lipophilic and thermosensitive, with a significant stability problem in an acidic solution [8]. Inclusion complexation with native or derived cyclodextrins is thought to be a practical technique for overcoming the stability and solubility issues of both agents [9].

On the other hand, many strategies have been tried successfully to regulate the gastrointestinal absorption of solid dosage forms. In this concern, alginate mucoadhesive properties have been able to delay the residence time and to slow down drug metabolism [10]. There is increasing interest in the preparation of beads from alginate gel because it is safe and can encapsulate medications. Furthermore, the drug-loaded alginate beads are frequently exposed to extra coating with chitosan. Compared to a single-unit preparation, the advantages of a gastro-retentive hydrogel beads system consisting of a multiple-unit particulate system are apparent [11].

In this study, we proposed a combined OMP/CURC oral dosage form loaded to alginate hydrogel beads coated with chitosan (CS). The study attempted to formulate, characterize, and select the best OMP/CURC alginate beads and assess their efficacy in healing experimental PUD in mice. 

## 2. Results and Discussion 

### 2.1. Phase-Solubility Experiments 

**Figure 1:**
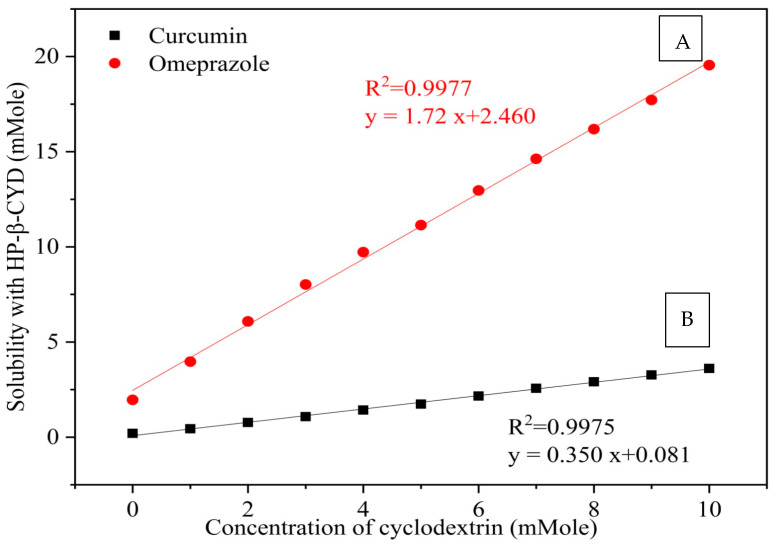
Phase solubility diagram of (**A**) CURC with HPR-β-CYD, (**B**) OMP with HPR-β-CYD systems using water at 25 °C. HPR-β-CYD: hydroxypropyl-β-cyclodextrin, OMP: omeprazole, CURC: curcumin.

The equilibrium phase solubility diagrams of CURC and OMP allowed for the determination of the stoichiometry of each drug inclusion complex with HPR-β-CYD individually (Figure 1). As shown in the graphs, the aqueous solubility of either CURC or OMP increases linearly as a function of the cyclodextrin concentration over the studied range. This linearity indicates that an AL-type solubility curve with no complex precipitation has been detected. The slope values are (0.350 and 1.72) for CURC and OMP curves, respectively, and since each of these values is less than 1, it is assumed that the increase in the solubility observed in each system is due to the formation of a 1:1 molar complex (Higuchi & Connors, 1965). It was found that the solubility of CURC increased by about 18.4-fold, whereas that of OMP reached 9.9-fold in the presence of 10 mM HPR-β-CYD.

Finally, the apparent stability constants of each drug–cyclodextrin complex were calculated, and their values are 6.6577 and 0.972 for CURC and OMP complexes, respectively. The solubility data of CURC are consistent with those reported previously [12].

### 2.2. Characterization of Inclusion Complexes

#### 2.2.1. Characterization of HPR-β-CYD/OMP Inclusion Complexes

The FTIR spectra were applied to confirm the formation of the HPR-β-CYD/OMP inclusion complex. This was investigated by recording the changes in the guest molecule’s distinctive bands, the absence or decrease in the intensity of peaks, the wave number change, or the emergence of additional bands that may suggest complex formation [13].

**Figure 2:**
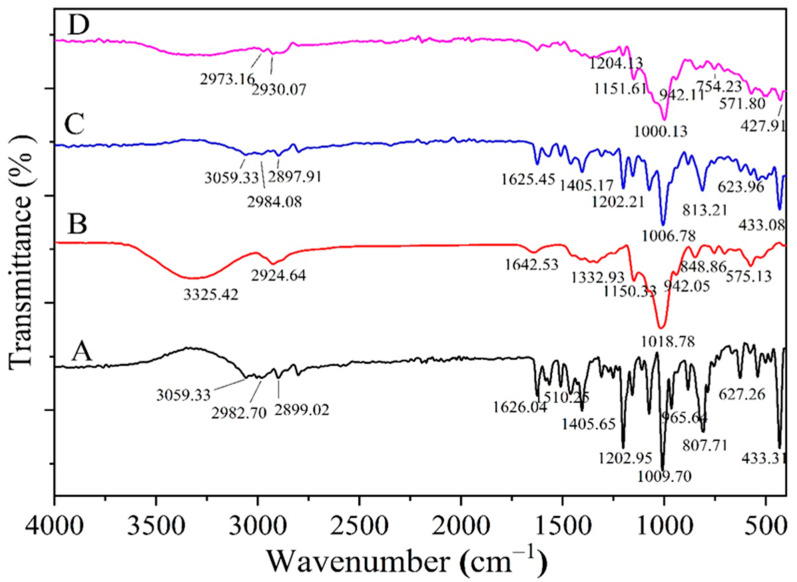
FTIR of (**A**) free OMP, (**B**) HPR-β-CYD, (**C**) HPR-β-CYD-OMP physical mixture, and (**D**) HPR-β-CYD/OMP inclusion complex. HPR-β-CYD: hydroxypropyl-β-cyclodextrin, OMP: omeprazole.

The FTIR spectra of OMP, HPR-β-CYD, the physical mixture of OMP and HPR-β-CYD, and the HPR-β-CYD/OMP inclusion complex are shown in Figure 2A,D. OMP displayed its characteristic bands (Figure 2A), especially the C = N bond stretching vibration bands at about 1626 cm^−1^, the N-H bending at about 1405 cm^−1^, and the asymmetric C-O-C stretching bands at about 1203 cm^−1^. These described OMP spectral features were distinguishable from those obtained from the HPR-β-CYD FTIR spectrum (Figure 2B). The FTIR of the OMP and HPR-β-CYD physical mixture was additive, with an observed reduction in the peak intensities of both compounds due to their dilution in their mixture (Figure 2C).

After the formation of the HPR-β-CYD/OMP inclusion complex, we observed a significant reduction in the band intensities corresponding to OMP in comparison with the intensities of the same bands acquired from the physical mixture (Figure 2C,D). Bands at about 1625, 1405, and 1202 cm^−1^ in the physical mixture were reduced significantly in the FTIR spectrum of the inclusion complex. Such modifications of the defining bands of the OMP functional groups demonstrate the profound implantation of OMP into the HPR-β-CYD cavity to produce the HPR-β-CYD/OMP inclusion complex. 

**Figure 3:**
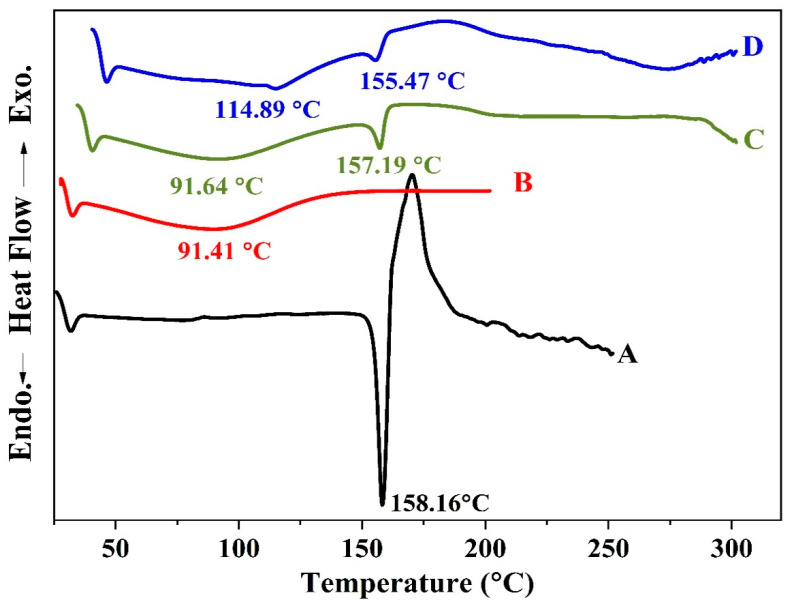
DSC spectra of (**A**) free OMP, (**B**) HPR-β-CYD, (**C**) HPR-β-CYD-OMP physical mixture, and (**D**) HPR-β-CYD/OMP inclusion complex. HPR-β-CYD: hydroxypropyl-β-cyclodextrin, OMP: omeprazole.

Other evidence for the formation of the inclusion complex is the comparison between the DSC thermograms of OMP, HPR-β-CYD, the physical mixture of OMP and HPR-β-CYD, and the HPR-β-CYD/OMP inclusion complex (Figure 3A–D). Concerning the OMP complexation, the endothermic peaks at 157.19 °C and 155.47 °C correspond to the OMP fusion in the drug–cyclodextrin physical mixture and the OMP-HPR-β-CYD kneaded product, respectively. However, the OMP-HPR-β-CYD kneaded product manifests a marked reduction in the drug enthalpy value relative to that observed in the physical mixture thermogram, where the drug ΔH value in the complex is –7.94, ΔH % is (7.05%) compared to –13.76, and ΔH % is 12.23%. Such a reduction in drug enthalpy in the solvent-processed product relative to the physical mixture suggests at least partial OMP-HPR-β-CYD complexation in the solvent-kneaded OMP-HPR-β-CYD preparation.

#### 2.2.2. Characterization of HPR-β-CYD/CURC Inclusion Complex

**Figure 4:**
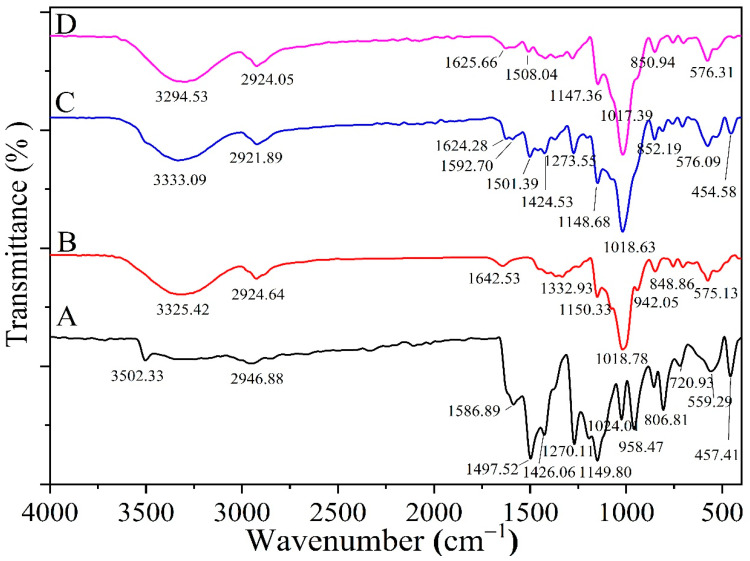
FTIR of (**A**) free CURC, (**B**) HRP-β-CYD, (**C**) HRP-β-CYD-CURC physical mixture, and (**D**) inclusion complex of HRP-β-CYD/CURC. HPR-β-CYD: hydroxypropyl-β-cyclodextrin, CURC: curcumin.

A comparison between the FTIR spectra of CURC, HPR-β-CYD, the CURC-HPR-β-CYD physical mixture, and the inclusion complexes enabled the deduction of the formation of the inclusion complex (Figure 4A,D). CURC characteristic bands were observed at about 1500 and 1590 cm ^−1^ the CURC and CURC-HPR-β-CYD physical mixture (Figure 4A,C), with an expected reduction in intensity in the physical mixture due to the dilution of the ingredients. CURC characteristic bands were reduced significantly upon the formation of the CURC-HPR-β-CYD inclusion complex (Figure 4C,D). The intensities of CURC characteristic bands at about 1501 and 1592 cm^−1^ decreased sharply in the complex spectrum in comparison with those observed in the physical mixture. This decreased intensity for the inclusion complex may be attributed to the development of the inclusion complex between HPR-β-CYD and CURC. 

**Figure 5:**
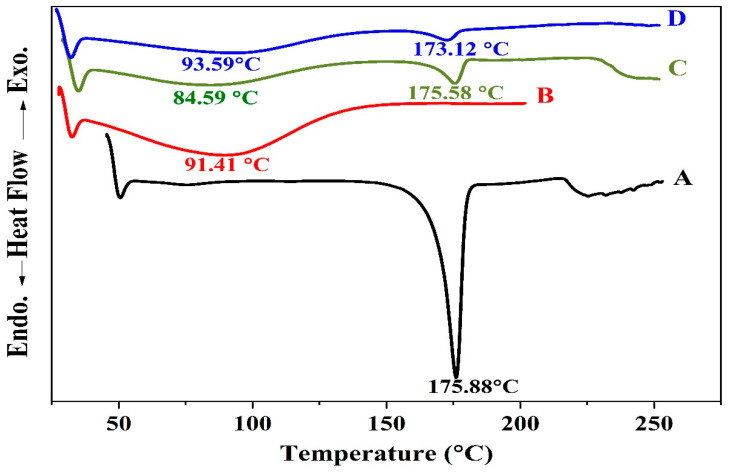
DSC spectra of (**A**) free CURC, (**B**) HPR-β-CYD, (**C**) HPR-β-CYD- CURC physical mixture, and (**D**) HPR-β-CYD/CURC inclusion complex. HPR-β-CYD: hydroxypropyl-β-cyclodextrin, CURC: curcumin.

The comparison of the thermograms of CURC, HPR-β-CYD, the CURC-HPR-β-CYD physical mixture, and the inclusion complexes supported the confirmation of the formation of the inclusion complex (Figure 5A,D) [14].

The CURC-HPR-β-CYD physical mixture thermogram exhibits an endothermic peak at 175.58 °C, representing the drug’s presence in the sample. In comparison, the thermal analysis of the corresponding solvent-kneaded product displays a shifted endothermic event at 173.12 °C, with a pronounced reduction in the drug enthalpy. Such data reveal a considerable loss of drug crystallinity and the formation of the drug-HPR-β-CYD complex that occupies a significant part of the sample. Compared to the solvent evaporation method [14].the solvent-kneading technique manifests only a slight enhancement of the complex formation; nevertheless, it is more practical in terms of time-saving and the ease of solvent elimination.

### 2.3. Formulation and Optimization using Two-Level Factorial Design

The benefits and adaptability of the factorial design facilitate a better understanding of the complexity of generating formulation development. Several independent factors define the number of needed trials, the responses for each experiment are determined, and multiple regression analysis is conducted. The current design had three variables and two experimental levels. All formulation trials were formulated and assessed for the various effects. 

#### 2.3.1. Beads Diameter

A vernier caliper was used to measure hydrogel beads with diameters of 1.5–2.6 mm, as shown in Table 1.

#### 2.3.2. Swelling Behavior of the Beads

Table 1 displays the beads’ swelling behavior at pH 1.2. The hydrogel beads swelled, causing beads with a modest amount of alginate to burst after just 2 h. The ion exchange mechanism takes place between the Na^+^ ion content in the buffer and the Ca^2+^ ion content in the cavity of Ca-alginate beads [15].The same remarkable response of alginate beads was also found through their swelling investigation with varying doses. The dry beads’ swelling behavior is mostly attributable to hydrating hydrophilic groups of alginate and CS [16].

#### 2.3.3. Drug Entrapment Efficiency (EE%)

The formulated beads were used to determine the drug concentrations by the measurement of UV absorbances at 304 and 424 nm for OMP and CURC, respectively. The measurement method validation parameters—selectivity, linearity, range, limits of detection, and quantitation and repeatability—were confirmed. The beads matrix had negligible spectrophotometric absorbance at 304 and 424 nm, thus indicating selectivity regarding matrix components. The method was linear in the range of 4 to 14 µg.mL^−1^, corresponding to 40–140% of the working concentration. The regression coefficients for the OMP and CURC were 0.9994 and 0.9997, respectively. Detection and quantitation limits were calculated from the regression line intercept standard deviation and response slope and were found to be equal to 0.38 and 1.14 µg.mL^−1^ for OMP, respectively, and 0.29 and 0.87 µg.mL^−1^ for CURC, respectively. The beads content assay showed good repeatability, as follows (expressed as the average assay; relative standard deviation %RSD): for OMP, an average of 101.1% and %RSD of 0.42%, while for CURC, an average of 102.0% and %RSD of 0.76%. The estimated values of EE% are shown in Table 1, revealing a higher entrapment efficiency. The more porous nature of CS-coated alginate beads may account for their greater loading efficiency. Since the Ca^+2^ and NH_3_^+^ of CS compete, they can react with the alginates -COO^−^.

The diameter range was 1.5 mm to 2.6 mm, the observed swelling minimal value was 400%, and the maximum value was 800%, while the EE% ranged from 60.85% to 87.44%. The effect of various factors and the responses of drug-loaded formulations are discussed separately in Table 1.

### 2.4. Development of Optimization Models

The values of the responses were optimized by using criteria based on the feasibility of a proposed solution. The numerical analysis component of the Design Expert® software (version 11). was responsible for the generation of all values. Developing a formulation of complex drug-loaded beads based on the concentration of the variables proposed by an overlay plot was used to determine the model’s accuracy. The optimal formulation (F8) was examined for a variety of responses, including EE%, bead size (mm), and swelling (%). The observed and projected responses were compared, and the error percentage was calculated (Table 1). The optimal formula of the complex medicines (F8) had an EE% of 87.44 ± 1.88%, swelling of 800 ± 6.2%, and bead size equal to 2.6 ± 0.24 mm. An observed reduced percentage of errors indicates that the developed model optimally fit for the two-level factorial design.

#### 2.4.1. Model Fit Report Describing the Impact of Sodium Alginate

**Figure 6:**
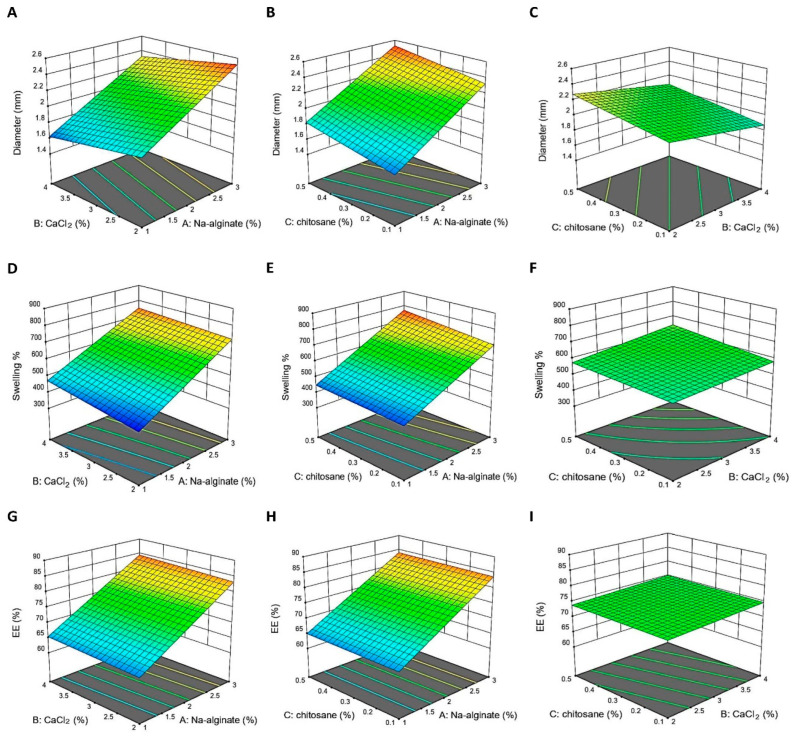
Nine response surface graphs demonstrating the influence of the variables. (**A**–**C**) demonstrate different variables’ effect on the diameter. (**D**–**F**) show the effect of different variables on the tested swelling %. (**G**–**I**) show various variables’ effects on the % entrapment efficiency (%EE) of beads.

Upon increasing the amount of sodium alginate, the diameter measurement showed increasements (Figure 6A,B) similar to those in a previous study [17]. Model graphs revealed that sodium alginate substantially affected the swelling % measurement in the formulations (Figure 6D,E). Upon increasing the sodium alginate concentration, the formulations’ swelling % increased, as demonstrated by Deshmukh et al., who noted a correlation between increasing diameter and swelling. According to model graphs, sodium alginate significantly impacts the EE% of the formulations. Figure 6G,H demonstrate that as the concentration of sodium alginate grew, so did the EE% of the formulations; this corroborates the findings of one research group. Similar outcomes were reported previously [18]. When sodium alginate was increased; the EE% in all formulations increased significantly.

#### 2.4.2. Effect of Calcium Chloride (CaCl_2_)

Upon the rise in the CaCl_2_ concentration, the surface of the responses moves from a larger diameter to a smaller one, as demonstrated by the model graphs in Figure 6A,C. This is readily apparent in the model plots of the figures. The increasing content of CaCl_2_ caused formulations to expand more (Figure 6D,F). At a pH of 1.2, the maxi-mum swelling ratios increased with an increasing CaCl_2_ concentration, and beads made in low CaCl_2_ solutions disintegrated earlier than those prepared in CaCl_2_ solutions of other concentrations. The calcium ion concentration may influence the cross-linking density of beads during production [19]. As in Figure 6H,I, the EE% was in-creased when the CaCl_2_ concentration was enlarged, since the crosslinking reaction was superior [20].

#### 2.4.3. The Effect of Coating the Beads with Chitosan

The higher CS concentration caused the formulations’ diameter to expand [21]. As depicted in Figure 6C, the elevation in the formulation diameter can be explained by the concomitant rise in the bead diameter, which suggests the production of a thicker chitosan layer. The swelling index at a pH of 1.2 increased considerably with the concentrations of CS, while in essential circumstances, the CS-coated alginate beads displayed the highest extent of swelling due to ionized carboxylic groups that are very hydrophilic. Therefore, CS-coated alginate beads may protect the medication from the harshly acidic environment of the stomach. The higher CS concentration causes formulations to expand more (Figure 6F) [21].

In Figure 6I, the lower concentration of CS caused a decrease in EE%, which may be attributable to the effects of the CS content on the formulation diameter and EE% [22]. A rise in the concentration of CS in the coagulation solution results in the production of a denser membrane due to an increase in the number of alginate-CS ionic bonds, which decreases the porosity of the gel structure and causes an increase in drug entrapment simultaneously. It may result upon the firmer and more compact arrangement of the formulated CS-coated alginate beads, which prevents drug diffusion during encapsulation.

### 2.5. Selection of the Optimized Formulation

**Figure 7:**
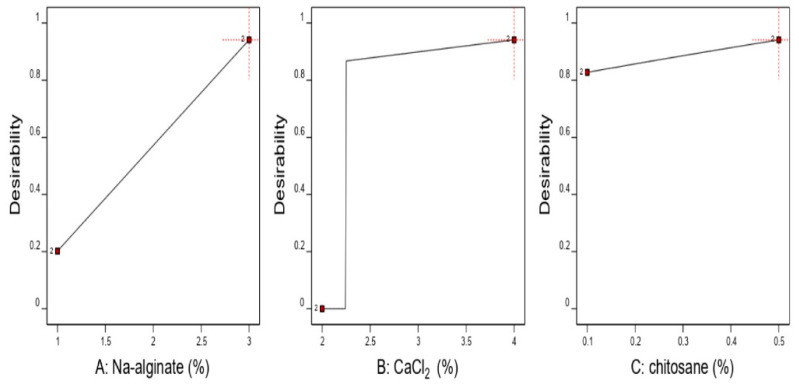
Effect of different independent variables on the selected optimum conditions.

**Figure 8:**
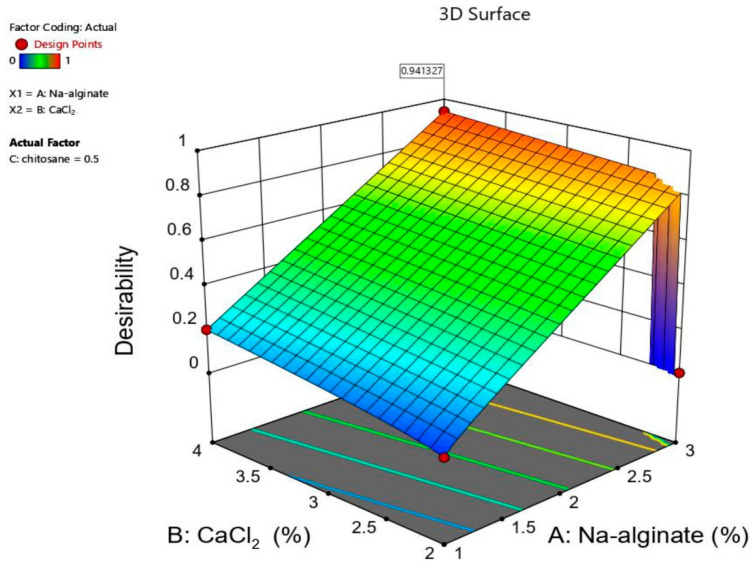
3D surface plot showing the desirability function for the optimum formulation predicted by Design-Expert® software (version 11).

The numerical optimization of design-Expert® software (version 11) enabled the selection of the optimized formula. The optimal formulation was predicted through the mathematical modeling and desirability function calculations (Figure 7). The desirability function was found to increase by increasing the method three variables (Figure 7 and Figure 8). A sharp, steep decrease in the desirability function was noticed at 3% of sodium alginate and 2% of CaCl_2_. This decrease is justified by the settings of the beads diameter optimization criteria in the desirability function calculation, which was defined to be in the range of 1.5–2.6 mm. The beads diameter exceeded the specified range at 3% of sodium alginate and 2% of CaCl_2_, causing the desirability function to fall sharply to zero to make the noticed steep decrease in the desirability function plot. 

The predicted optimum conditions were found to result from using the maximum value of the variables, CaCl_2_%, sodium alginate%, and CS%, as depicted in the desirability plot. This resulted in a desirability value equal to 0.941 (Table 1). According to this prediction, the optimum formulation parameters for sodium alginate, CaCl_2_, and the CS polymer content were 3%, 4%, and 0.5%, respectively (Figure 8). These values corresponded to a formulation with a predicted diameter of 2.60 mm, % swelling of 800.00, and EE of 87.44%.

#### 2.5.1. Morphology for the Optimal Formulation of the Hydrogel Beads

**Figure 9:**
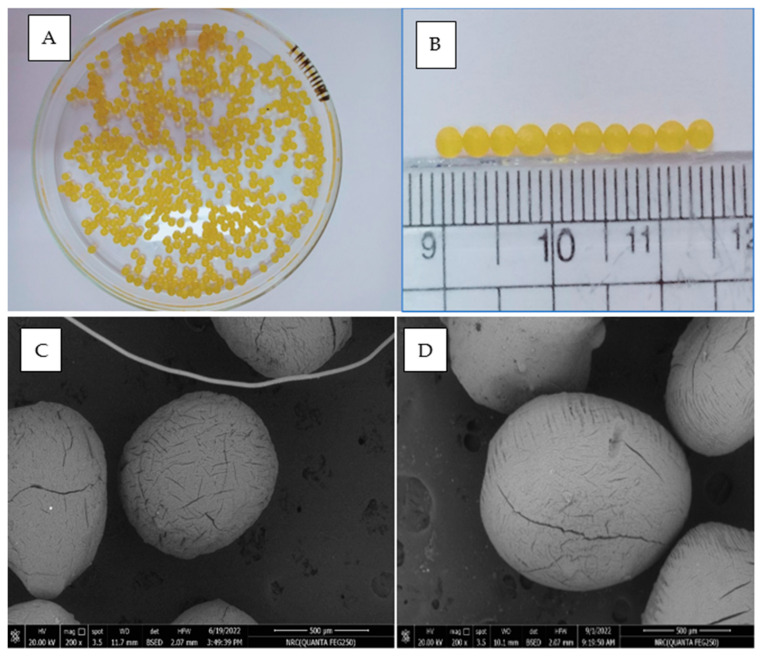
Digital photos for (**A**) the particle size of the optimal formula, (**B**) the optimized formulation hydrogel beads, (**C**) scanning electron micrography for blank beads of the unloaded drug, and (**D**) drug-loaded beads.

The smooth bead surface significantly prolonged the releasing time [23]. As observed in Figure 9, the form and surface of beads in their wet state were spherical in shape and smooth. The diameter was around 2.60 mm. Figure 9A,B depict the SEM images of the blank and the drug-loaded hydrogel beads, respectively. The microstructure shows the partial collapse of the polymeric network, which manifests as surface fissures. Dehydrating the polymeric beads throughout air drying increases the risk of fractures. Figure 9C depicts SEM micrographs of empty beads, which were more uniform and smaller than the drug-loaded beads shown in Figure 9D. 

The drug-loaded beads showed a regular surface morphology. The SEM outcomes were reported previously [24]. The authors documented similar observations while studying alginate-CS microspheres. Figure 9D shows the spherical shape of the drug-loaded beads.

#### 2.5.2. FTIR for Optimized Formula Beads

**Figure 10:**
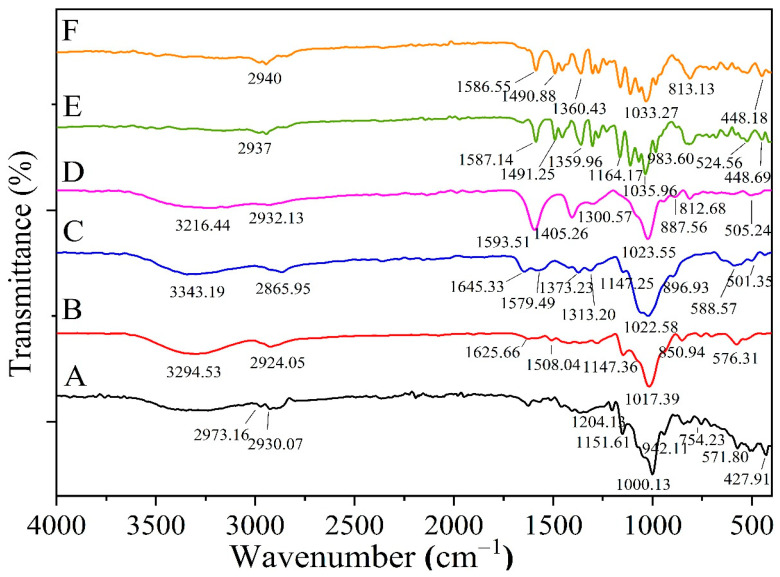
FTIR spectra of (**A**) HPR-β-CYD/OMP inclusion complex, (**B**) HPR-β-CYD/CURC inclusion complex, (**C**) Chitosan, (**D**) Sodium alginate, (**E**) Optimal form of the mixture, and (**F**) Optimal formula of the beads.

Figure 10 depicts the infrared spectroscopic study of pharmaceuticals, OMP-complex, CURC-complex, CS, sodium alginate, and optimized formula beads. Based on FTIR measurements (Figure 10), there is no discernible chemical change between medicines and beads. Specifically, the change in the bands at 1587.14, 1491.25, 1035.96, and 2937 cm^−1^, the removal of the band at 1164.17 cm^−1^, and the distinctive peak of the CS indicate the creation of a polyelectrolyte complex between alginate and CS. For OMP- and CURC-loaded beads, the typical absorptions of OMP and CURC are marked by stronger signals from the beads’ components, indicating that OMP and CURC molecules were absorbed into the polymeric network of beads. At an analogous wave number, the peaks were evident in the optimized spectrum of beads. Due to the possibility of the polymer matrix molecular dispersion, the drug peak was not particularly prominent within the formulation. According to the FTIR results, the polymer and the medication were not chemically incompatible. The FTIR analysis demonstrated that the combination medication complex was effectively loaded onto beads.

#### 2.5.3. DSC Thermograms for the Optimized Formulation of the Coated Beads

**Figure 11:**
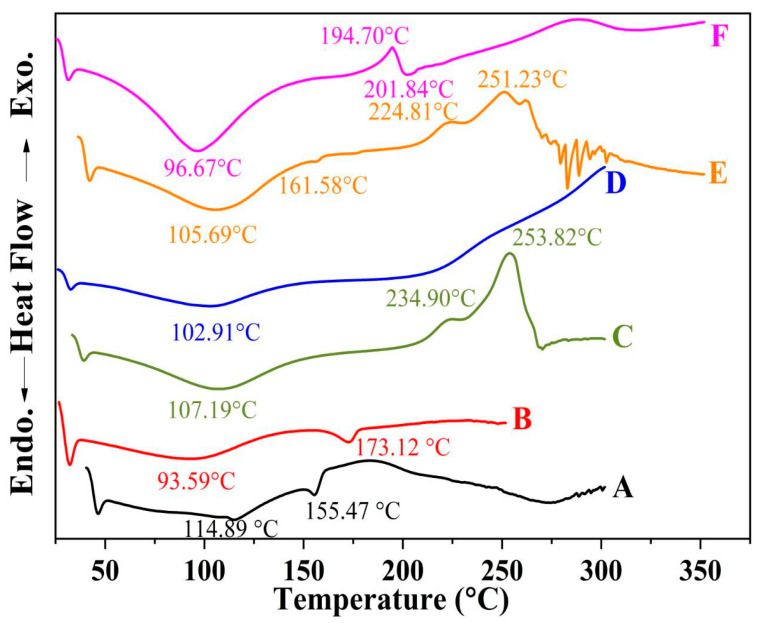
DSC spectra of (**A**) HPR-β-CYD/OMP inclusion complex, (**B**) inclusion complex for HPR-β-CYD/CURC, (**C**) sodium alginate, (**D**) chitosan, (**E**) optimized formula physical mixture, and (**F**) optimized beads formula.

The medicine DSC thermograms (OMP-complex and CURC-complex), sodium alginate, CS, and formula-optimized beads are depicted in Figure 11. The DSC results also show that there was not any drug polymer interaction during the manufacturing process. The revised formulation displayed a broad polymer matrix melting peak at its thermal peak. This points to the dual drug undergoing some metamorphosis into an amorphous state. The medication being trapped in the polymer matrix could cause the observation.

#### 2.5.4. In Vitro Release and Kinetic Release Study

**Figure 12:**
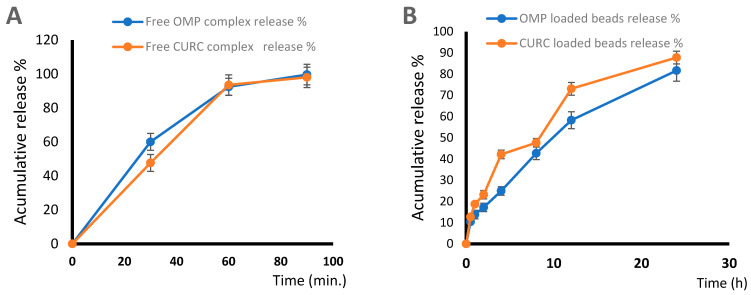
Release profiles of (**A**) Free OMP complex, free CURC complex, and (**B**) OMP beads and CURC beads in 0.1 HCl. OMP: omeprazole, CURC: curcumin.

In the first hour following the administration of the free drug complex, 95% of OMP and 98% of CURC were released (Figure 12A). This is unacceptable for medications that require a delayed release in the stomach. Figure 12B depicts the medication release from hydrogel beads in various GIT fluid simulations. The initial drug release from hydrogel beads was minimal. Only 23.19% (for CURC) and 17.19% (for OMP) were released after 2 h, and 73.09% (CURC) and 58.26% (OMP) were released after 12 h, but after 24 h, about 87.81% (CURC) and 81.67% (OMP) had been released. The film’s integrity is compromised as ionization occurs and the medication is released. At pH 1.2, the membrane CS coating disintegrated, exposing the hydrogel beads. Following the swelling and erosion of the polymer matrix, the medication was released.

#### 2.5.5. Bio-Adhesion Test

**Figure 13:**
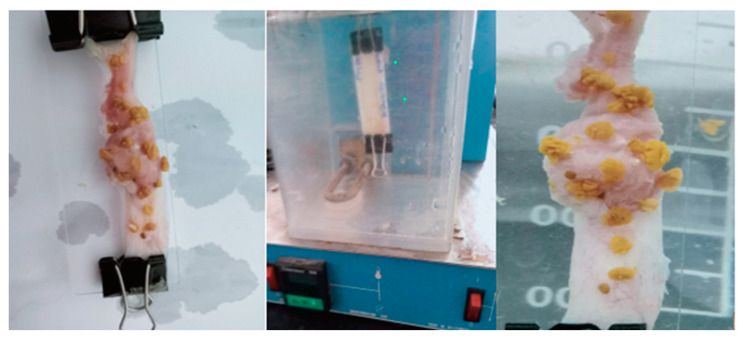
In vitro bio-adhesion test for the formulated hydrogel beads.

The outcomes of the bio-adhesion tests on beads contain two medicines (OMP and CURC), as shown in Figure 13. Initially, there were 20 beads in the mucosa; after 30 min of flow with 0.1 N HCl solution, there were 20 beads (100%); after 2 h, there were 19 beads (95%) remaining; after 6 h, there were 17 beads (85%). The bio-adhesion capabilities of the beads are considered the most important parameter, since they are directly related to the efficiency of the system in targeting the drug to the gastric and upper duodenal regions. These studies demonstrate that the combination of OMP and CURC in beads has bio-adhesive characteristics.

### 2.6. In Vivo Treatment Efficacy

#### 2.6.1. Assessment of Macroscopic Injury of Gastric Tissues

**Figure 14:**
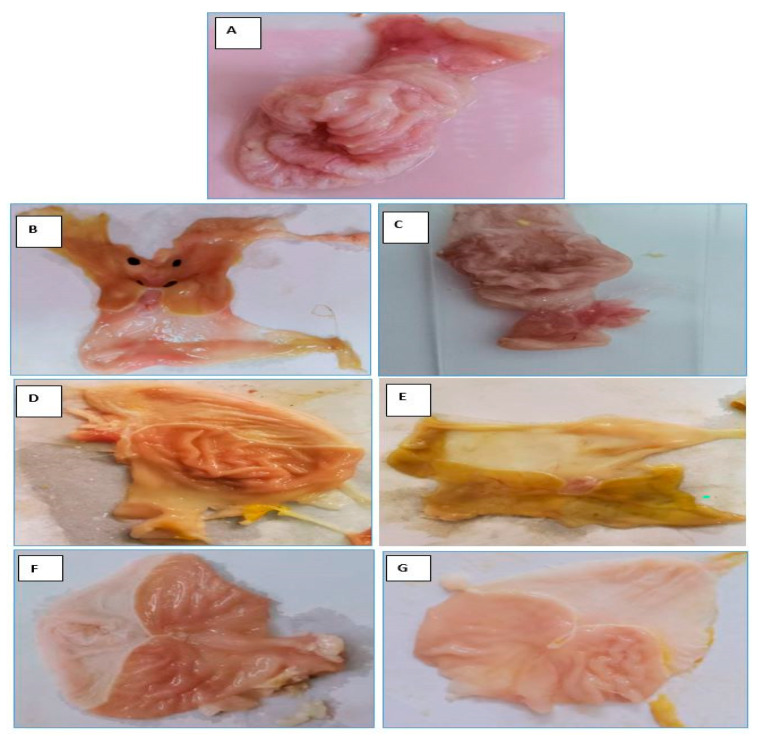
Gross appearance of the mucosal layer in stomachs of indomethacin-induced models: Normal group (**A**), PUD control group (**B**), blank beads-treated group (**C**), free OMP-treated group (**D**), CURC-only-loaded beads-treated group (**E**), OMP-only-loaded beads-treated group (**F**), and OMP/CURC-loaded beads-treated group (**G**). OMP: omeprazole, CURC: curcumin, PUD: peptic ulcer disease.

Figure 14 depicts a macroscopical inspection of the rat stomachs. The stomachs of the normal control group (Figure 14A) displayed a healthy mucosal lining, no inflammation, and no blood loss or hyperemia. In contrast, groups 2 and 3 showed substantial stomach mucosa damage (Figure 14B,C). When given orally, indomethacin (50 mg/kg) caused extensive hemorrhagic abrasions, most of which were located on the stomach glandular mucosa segment, and a few or none were located in the antrum. Several ulcer patches of varying sizes and shapes within the stomach mucosa were dark crimson and covered the bulk of the stomach. The stomach mucosa showed exceptional hyperemia. In the stomachs of animals in PUD + free OMP (Figure 14D), the number of ulcers was dramatically reduced. Discontinuous lesions developed in the stomach mucosa, and perforation did not occur in any rat. The stomach mucosa showed exceptional hyperemia. In the stomachs of animals in PUD + CURC-only beads (Figure 14E), the number of ulcers was dramatically reduced. Discontinuous lesions developed in the stomach mucosa, and perforation did not occur in any rat. While treatment with OMP-only beads resulted in a milder ulceration reaction (Figure 14F), fewer tiny red lesions were observed on the gastric mucosa, and the inhibitory effect was more pronounced than it was in the free OMP treatment group. Interestingly, therapy with OMP/CURC-loaded beads coated with chitosan formula (Figure 14G) resulted in a reasonably normal-appearing stomach, with very few ulcers and no evidence of inflammation or hemorrhage. The treated animals were healthy during the experiment and had typical activity levels. Based on the acquired data, it is evident that healing animals with the formed beads provided a superior curative effect to PUD induced by indomethacin compared to treatment with free OMP. This can be explained by the prolonged duration of drug release and the maintenance of retaining an optimal medication concentration for an extended period. Using OMP/CURC beads dramatically enhanced protection against stomach ulcers compared to the use of OMP beads alone. This is mostly attributed to the gastroprotective properties of CURC.

#### 2.6.2. Histological Examination

**Figure 15:**
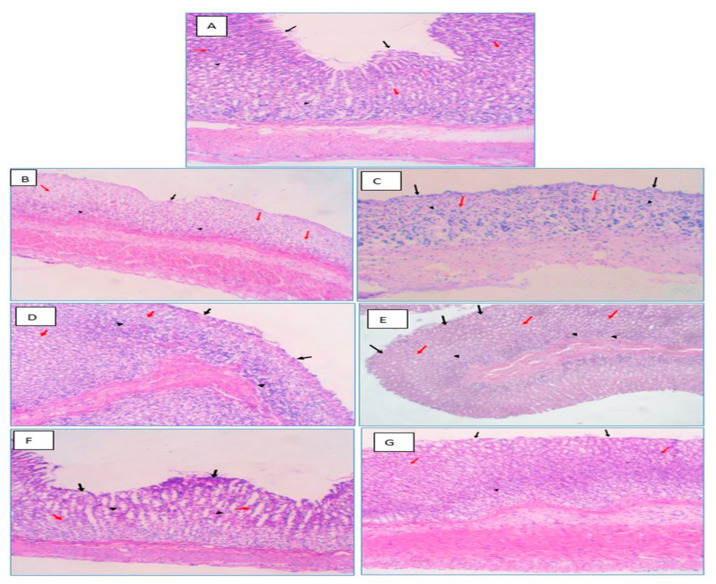
Histological pictures of stomach specimens stained with hematoxylin and eosin from the indomethacin-induced ulcer model in rats: Normal control group (**A**), disease control group (**B**), blank beads-treated group (**C**), free OMP-treated group (**D**), CURC-only-loaded beads-treated group (**E**), OMP-only-loaded beads-treated group (**F**), and CURC/OMP-loaded beads-treated group (**G**). (**A**) Uniform gastric tissue showing regular epithelial cell covering (Black arrow), overlying lamina propria showing few chronic inflammatory cell infiltrates (Arrowheads), and uniform mucous-secreting gastric glands (Red arrows). (**B**) There is significant ulceration in the surface epithelium (Black arrows). The lamina propria is chronically lymphoplasmacytic (Black arrowheads). There are several foci of atrophic glands (Red arrows). (**C**) There are eroded surface linings (Black arrows). There is significant atrophy in mucosal glands (Red arrows) and foci of chronic lymphoplasmacytic infiltrate involving lamina propria (Arrowheads). (**D**) There is still an eroded surface epithelium (Black arrows) and the partial recovery of the mucosal glands (Red arrows), with a reduction in chronic inflammatory cell infiltrate (Black arrowheads). (**E**) There is still an eroded surface epithelium (Black arrows) and the partial recovery of the mucosal glands (Red arrows), with a reduction in chronic inflammatory cell infiltrate (Black arrowheads). (**F**) There is some restoration of the surface epithelium (Black arrows) and complete recovery of the mucosal glands (Red arrows), with a reduction in chronic inflammatory cell infiltrate (Black arrowheads). (**G**) There is a restoration of the surface epithelium (Black arrows) and complete recovery of the mucosal glands (Red arrows), with a significant reduction in chronic inflammatory cell infiltrate (Arrowheads) in the OMP/CURC-treated rats (H&E, 100×). OMP: omeprazole, CURC: curcumin.

Histopathologic evaluation was performed blindly to detect the pathological alterations in the gastric mucosa and other layers of the animal groups. Figure 15 and Table 2 depict the histological effect of several therapies on a PUD model caused by indomethacin. The histology of the stomachs of normal control rats reveals a normal microscopic appearance and architecture and normal glandular structures, while no abnormalities were detected. In the upper part of the stomach glands, the parietal cells have eccentric nuclei and pale cytoplasmic eosinophilic vacuoles (Figure 15A). In contrast, the result demonstrates an intense inflammatory response, characterized by submucosal edema and localized mononuclear infiltrating leukocytes in the lamina propria, muscularis mucosa, and submucosal layers of the gastric tissue for the indomethacin-induced PUD control group (2). Arteries in submucosa were constricted, and the muscular coat showed hypertrophy and was somewhat hyalinized. These findings demonstrated the ulcer’s acute phase and significant inflammation (Figure 15B). Similar observations demonstrated an acute phase of the ulcer and a significant inflammatory response in the stomach tissue of the PUD + blank beads group (Figure 15C). The stomach tissue of the PUD + free OMP group showed a moderate inflammatory response (Figure 15D). The stomach tissue of the PUD + CURC-only beads group showed a moderate inflammatory response (Figure 15E). The severity of the pathologic features was reduced in the PUD + OMP only beads group (Figure 15F), which displayed modest quantities of leukocytes infiltrating into the submucosa and muscularis mucosa. In Group 7, " PUD + OMP/CURC" group, the mucosa was greatly preserved. Stomach tissue specimens exhibited consistent epithelial and glandular morphology, organization, and excellent tissue architecture. In tissue slices, only minimal leucocytic inflammatory cells were observed (Figure 15G). 

## 3. Materials and Methods

### 3.1. Materials

Omeprazole, CURC, hydroxypropyl-β-cyclodextrin (HPR-β-CYD), sodium alginate, and chitosan were purchased from Sigma-Aldrich (St. Louis, MO, USA). Hydrochloric acid (HCl), methanol, and ethyl alcohol (99%) were obtained from El-Nasr Pharmaceutical Co. for (Cairo, Egypt). Anhydrous calcium chloride (Fine GRG 90%) was supplied by Fisher Scientific (Fairlawn, NJ, USA).

### 3.2. Methods

#### 3.2.1. Phase Solubility Studies

Drug solubility assessments for CURC and OMP were carried out separately using the phase solubility technique described by Higuchi and Lach [25].

Excess amounts of each drug were added to a series of aqueous solutions containing different concentrations of HPR-β-CYD (1–10 mMole) (HPR-β-CYD used as a solubilizing agent to increase the solubility of CURC and OMP) [26].The mixtures were ultrasonically irradiated for 30 min and then shaken mechanically for 5 days at 25 °C. After equilibrium, the solutions were filtered through a Millipore filter (0.45 µm). Aliquots of the filtrate were diluted and analyzed spectrophotometrically at 424 nm and 304 nm to determine the drug concentrations of CURC and OMP, respectively. An apparent stability constant was calculated from the straight line diagram according to the following Equation (1) [27].

KC = Slope/intercept (1-slope)(1)

#### 3.2.2. Preparation of the HRP-β-CYD/Drug Inclusion Complex

A modified solvent kneading technique was used to prepare CURC and OMP inclusion complexes with HPR-β-CYD, assuming a 1:1 drug-to-cyclodextrin molar ratio [28].Accurate weights of either CURC or OMP were physically blended with HPR-β-CYD in a glass mortar adopting the geometric dilution technique. A solvent mixture of equal portions of chloroform, acetone, ethanol, and water (1:1:1:1) was added gradually with continuous stirring until a clear homogenous solution was formed. The obtained solution was then placed in a fume hood and stirred magnetically at 25 ± 1 °C for 24 h to permit the complete evaporation of the solvent. When most of the solvent was evaporated, the resulting smooth paste was thoroughly kneaded using a pestle. The product was placed in a circulating hot air oven overnight at 50 °C to allow for the complete dryness of the sample. Finally, a sieve with 80-mesh openings was used to screen the sample. The resulting solid powder was kept protected from light under desiccation at 25 ± 1 °C [29].

#### 3.2.3. Characterization of Drug Complex-HPR-β-CYD

Fourier transform infrared (FTIR) and DSC were utilized to describe the inclusion complexes and confirm their formation in the dried drug/cyclodextrin preparation. FTIR spectroscopy was used as a means of defining the solid-state HPR-β-CYD/drug complex [30]. FTIR spectra were captured on a Nicolet 6700 FTIR spectrometer (Thermo Fisher Scientific, Waltham, MA, USA) with a scanning scope ranging from 4000 to 400 cm^−1^. Potassium bromide palettes were utilized for obtaining the spectra.

#### 3.2.4. Optimization of Formulation Variables

ANOVA with two-level factorial design (Design Expert^®^ (version 11.1.2.0, Stat-Ease Inc., Minneapolis, MN, USA)) was utilized to optimize the conditions for the manufacture of CS-coated alginate beads. Table 3 lists the independent and dependent variables. The experimental design was utilized to analyze the relationship between the independent variables and their responses and interactions to develop a valid model; it consisted of three variables and three responses. However, the dependent response factor variables measured were entrapment efficiency (EE%, Y1), degree of swelling (Y2), and particle size (Y3). The independent variables are alginate concentration (X1), CaCl_2_ concentration (X2), and CS concentration (X3). The findings were analyzed and confirmed in terms of statistically significant coefficients with R^2^ values over the entire experimental zone (Table 3). The formulation factors and the high and low levels of each variable were determined based on initial experiments and a literature review. The design matrix was provided in a coded form in Table 3.

#### 3.2.5. Preparation of Chitosan-Alginate Beads

The CS-coated alginate beads were formulated based on the procedure described previously in the literature, with some modifications [30,31]. First, sodium alginate (1–3% *w*/*v*) was dissolved in distilled water by stirring at 900 rpm at room temperature. The obtained solution was heated to 70 °C and mixed (900 rpm) before the complex was dissolved in it. Accurate weights of the already-prepared drug/HPR-β-CYD complex powder equivalent to 30 mg of OMP and to 20 mg of CURC were then dissolved in the alginate solution (Solution A). As a coagulation fluid, a homogeneous solution of CS (0.1–0.5%) and CaCl_2_ (2–4%) dissolved in 1% *v*/*v* aqueous acetic acid was utilized (Solution B). The alginate–drug/HPR-β-CYD complex mixture (solution A) was then extruded into solution B with mechanical stirring at 200 rpm at room temperature. The solution’s pH was subsequently adjusted to 5 ± 0.1 using 0.1 N sodium hydroxide, and the flow rate of medicines incorporated in the sodium alginate complex was maintained at 1 mL/min. The drug-loaded beads were formed, and the stirring was held for 30 min in the coagulation fluid. At the end of the procedure, the beads were filtered, washed, and air-dried [32,33].

### 3.3. Characteristics of the Beads

#### 3.3.1. Bead Diameter Measurement 

The diameter of the beads was determined by using a micrometer screw gauge, and the average values were taken for at least 10 beads [34].

#### 3.3.2. The Swelling Experiment 

The swelling of the beads was assessed via the immersing techniques described by Anal and Stevens (2005) [21]. The experiment was performed in a water bath shaker at 37 ± 0.5 °C. Pre-weighed dry beads were installed in 0.1 N HCl and permitted to swell. Beads were collected at a predetermined time after 2 h and wiped with filter paper to remove any excessive buffer. Weight changes of the collected beads were monitored regularly until equilibrium was reached. The degree of swelling was computed using Equation (2) [35].

% Swelling = (Wt. of swollen beads-Wt. of dry beads)/(Wt. of dry beads) × 100(2)

#### 3.3.3. Encapsulation Efficiency (EE%)

A direct technique was employed to evaluate the bead’s drug content. Briefly, 200 mg of hydrogel beads were weighed and finely ground in a glass mortar. The crushed beads were collected and dispersed in methanol (10 mL), after which the mixture was placed in a shaking water bath at 100 rpm at room temperature (37 ± 0.5 °C) for one day. A syringe equipped with a 0.45 µm filter was utilized for the filtration of the extract. In total, 1 mL of these samples was diluted with 10 mL of methanol, the concentrations for either CURC or OMP were assessed spectrophotometrically (Shimadzu, Kyoto, Japan) with reference to their respective analytical standard curve, and their concentrations were obtained. The encapsulation efficiency for OMP and CURC was calculated by using Equation (3) [36].

Drug encapsulation efficiency (EE)% = AQ/(TQ) × 100(3)

AQ and TQ are the actual and theoretical quantities of drugs in complex beads, respectively. The results were repeated three times.

### 3.4. Optimization Employing Two-Level Factorial Design

The optimal formula was selected depending on the response variables’ desirability and was checked physiochemically, in vitro and in vivo.

#### 3.4.1. SEM Analysis for Optimized Formula Beads

Hydrogel bead morphology and surfaces were examined using a scanning electron microscope (SEM) (JEOL, JSM-6380LV, Akishima Tokyo, Japan) [37]. Regarding the hydrogel samples, they were coated with gold and then scanned at 30 kV accelerating voltage before being glued on aluminum stubs with double sided adhesive tape for examination.

#### 3.4.2. FTIR Analysis for the Optimized Formula 

For FTIR, the samples were prepared by grinding the beads into a fine powder using a mortar and pestle. The sample was then submitted under an FTIR probe with a range of 400 to 4000 cm^−1^ and a transmittance of 95%. FTIR spectroscopy is used to determine the existence of interactions and identify modifications in the functional group composition.

#### 3.4.3. DSC Studies for the Optimized Formula 

DSC 60 Henrich Software is used for differential scanning calorimetry (DSC) investigations of the prepared drug-loaded beads. The dried bead weight for the DSC experiment ranged from 4 to 6 mg, and the weighed samples were placed in aluminum pans equipped with a sealed shut pinhole cover. All samples were evaluated under nitrogen flow with a scan rate of 10 °C per min and a temperature range of 25 to 300 °C. The DSC is used to determine the melting point, polymorphic alterations, and crystallinity changes and to identify any potential interactions between the drug, carrier, and adsorbent.

#### 3.4.4. In Vitro Drug Release from Optimized Formula Beads

A drug release investigation was conducted using a USP apparatus type II (Copley, England) at 100 rpm under sink conditions. To do that, 750 mL of 0.1 N HCl (pH 1.2) served as the release medium to simulate the acidic gastric environment. Samples of the release medium were withdrawn at specified time intervals up to 24 h. After each sampling, the withdrawn volume was compensated by a freshly prepared medium. Concentrations were measured at 304 and 424 nm for OMP and CURC, respectively, using an ultraviolet (UV) spectrophotometer, the Shimadzu UVPC UV-Vis spectrophotometer (Shimadzu, Japan).

#### 3.4.5. Bio-Adhesion Test

The stomach mucosa of rats was washed and immersed in a sterile solution of normal saline. Then, it adhered to the microscopic slide, with the mucosal side facing upwards. Microscopic slides were hung in a disintegration testing apparatus. The slides were made to go up and down in a water bath filled with deionized water with 0.1 N HCl solution to detect the drug in the release media resembling gastric fluid without enzymes in a fasting state, and the temperature was adjusted to be 37 °C. The volume of the solution in the water bath was adjusted so that, at the highest point of movement of the apparatus, the slides do not go out of the testing solution, and at the lowest point, they do not touch the bottom. This is carried out to make the movement of the testing solution in relation to the slide smooth and not turbulent. Before hanging the slide, the mucosal surface of the intestinal piece is irrigated with some of the testing media to simulate the real conditions. Twenty alginate beads loaded with OMP/CURC were affixed to the gastric mucosa, which was then put at room temperature for one minute and dripped with artificial gastric fluid. The number of beads remaining in the mucosa was then determined after 30 min, 2 h, and then 6 h [38].

#### 3.4.6. In Vivo Study for Optimized Formula Hydrogel Beads

Male Wistar rats with an original body weight in the range of 170–200 g were obtained from the animal house at the College. They were sheltered in typical requirements of relative humidity, temperature, and a normal light/dark cycle. Rats had free access to commercial pellets and water. The Suez Canal University Faculty of Pharmacy ethical committee approval number is 202009PHDA1. The animals were kept without food for 24 h but given free access to water. 

Each group, except for group 1, received an aqueous suspension of indomethacin in 1% sodium carboxymethylcellulose (CMC) at a dose of 50 mg/kg of body weight, 2 mL, for two consecutive days to cause PUD. The animals were then starved for 12 h before being randomized into seven experimental groups of five rats each. The rats received the medications once a day for 15 consecutive days.

(1) Vehicle control rats: received doses of CMC parallel to indomethacin.
(2) PUD control: received indomethacin but no medication.
(3) PUD + blank beads group.
(4) PUD + free OMP group (20 mg/kg; orally: intragastric route).
(5) PUD + CURC-only beads group (20 mg/kg; orally: intragastric route).
(6) PUD + OMP-only beads group (20 mg/kg; orally: intragastric route).
(7) PUD + beads loaded with CURC/OMP complex group (20 mg/kg, 20 mg/kg; orally: intragastric route).

On day 16, stomachs were checked macroscopically for hemorrhagic lesions. Microscopically, the number of ulcers per stomach and the severity of the ulcers were documented. The stomach was cut open, cleaned with ice cold saline, and blotted with filter paper. Ulcer projections were evaluated visually, and the specimen was fixed in 10% *v*/*v* formalin solution and stored for histological examination/evaluation [39].

In general, we compared the PUD + beads loaded with the CURC/OMP complex with the PUD rats treated with free OMP, CUR-only beads, and OMP-only beads to show the possible enhanced activity. Hence, any enhanced activity will belong to the additional CURC content.

#### 3.4.7. Histological Assessment of Rat Gastric Tissues

The isolated stomach tissue specimens were submerged in 10% formalin and then fixed in paraffin. The sections were colored with hematoxylin and eosin at a thickness of 4–5 µm. As described in the literature, microscope changes in the stomach mucosa were evaluated by the Sydney system [14]. Pathological changes in the specimens, such as chronic lymphoplasmacytic infiltration, neutrophils attacking the glands, areas of atrophying glands, and intestinal metaplasia, were examined and quantified. The intensity of the histopathological alterations was measured by utilizing the following four-point scale: (0) no lesions, (1) mild lesions, (2) moderate lesions, and (3) serious lesions.

### 3.5. Statistical Analysis

The results of the experiment were gathered and presented as the mean ± SD. An ANOVA test followed by Bonferroni’s test were performed to determine the statistical significance of differences between groups. Differences were determined at *p* < 0.05. 

## 4. Conclusions

The present work has indicated that oral therapy of indomethacin-induced PUD with the developed combination dual medicines (OMP & CURC complex) beads for 15 successive days markedly enhanced the healing of experimental stomach ulcer in rats compared to free OMP, CURC-only beads, and OMP beads alone. The anti-inflammatory characteristics and in vivo efficacy have been studied. The optimal formulation, F8, displayed the greatest swelling value at pH 1.2 due to it forming less network rigidity at low concentrations of sodium alginate 1%. At pH 1.2, the recorded profiles for drug release were in correlation with the largest swelling values (800.00 ±6.2) and highest EE% (87.44 ± 1.88). The in vitro results, performed in acidic media for 1 h following the administration of the free drug complex, were 95% release for OMP and 98% release for CURC. The initial drug release from hydrogel beads was minimal. Only 23.19% (CURC) and 17.19% (OMP) at the same media after 2 h and about 87.81% (CURC) and 81.67% (OMP) had been released after 24 h of sustained release. The bioadhesion test showed very good bioadhesion after 6 h (85%); this indicated that the drug remains for a long time in the site of action. The combination of the formulated OMP with CURC beads has shown synergistic efficacy in the healing of stomach ulcers compared to Free OMP, CURC-only beads, and OMP beads only, according to the histopathology test.

## Figures and Tables

**Table 1:** Various levels of independent variables in the different runs resulted in the dependent variable of formulations.

Run	SA (%)	CaCl_2_ (%)	Chitosan (%)	Diameter (mm)	Swelling%	EE%
F1	1	2	0.50	2.00 ± 0.32	408.00 ± 4.3	60.85 ± 1.01
F2	1	4	0.10	1.50 ± 0.08	455.56 ± 7.0	64.89 ± 3.62
F3	1	4	0.50	1.60 ± 0.16	488.24 ± 5.0	66.50 ± 0.94
F4	1	2	0.10	1.80 ± 0.14	400.00 ± 8.5	67.10 ± 1.95
F5	3	4	0.10	2.20 ± 0.08	708.09 ± 3.5	80.88 ± 0.93
F6	3	2	0.50	2.50 ± 0.21	743.14 ± 2.7	83.20 ± 0.80
F7	3	2	0.10	2.40 ± 0.08	700.00 ± 3.6	83.81 ± 1.73
F8	3	4	0.50	2.60 ± 0.24	800.00 ± 6.2	87.44 ± 1.88

Data are the mean ± SD, *n* = 3. SA: sodium alginate, EE%: entrapment efficacy %.

**Table 2:** Histopathological evaluation of stomach tissue sections.

		Scoring			
	Groups	Chronic Lymphoplasmacytic Infiltration	Neutrophils Attacking the Glands	Areas of Atrophying Glands	Intestinal Metaplasia
A	Group 1 (Normal)	0	0	0	0
B	Group 2 (PUD Control received no treatment)	2	0	3	0
C	Group 3 (PUD + blank beads)	2	0	3	0
D	Group 4 (PUD + free OMP)	2	0	2	0
E	Group 5 (PUD + CURC-only beads)	2	0	2	0
F	Group 6 (PUD + OMP-only beads)	2	0	1	0
G	Group 7 (PUD + OMP/CURC-loaded beads)	1	0	0	0

OMP: omeprazole, CURC: curcumin, PUD: peptic ulcer disease.

**Table 3:** Independent and dependent variables used in a two level factorial design.

Factors	Independent Variables		Low	High
X_1_	Na Alginate	%	1	3
X_2_	Ca Chloride	%	2	4
X_3_	Chitosan	%	0.1	0.5
Response				
Y_1_	Diameter	Mm		
Y_2_	Swelling	%		
Y_3_	Entrapment Efficiency (EE)	%		

## Data Availability

Data is contained within the article.
